# New insights from short and long reads sequencing to explore cytochrome *b* variants in *Plasmopara viticola* populations collected from vineyards and related to resistance to complex III inhibitors

**DOI:** 10.1371/journal.pone.0268385

**Published:** 2023-01-19

**Authors:** Semcheddine Cherrad, Benjamin Gillet, Julien Dellinger, Lalie Bellaton, Pascale Roux, Catalina Hernandez, Hervé Steva, Lauriane Perrier, Sébastien Vacher, Sandrine Hughes

**Affiliations:** 1 CONIDIA, Parc d’activités en Chuel, Quincieux, France; 2 Institut de Génomique Fonctionnelle de Lyon (IGFL), CNRS UMR 5242, Ecole Normale Supérieure de Lyon, INRAE USC 1370, Université Claude Bernard Lyon 1, Lyon, France; 3 CONIPHY, Parc d’activités en Chuel, Quincieux, France; 4 CJH SARL, La Brede, France; Youngstown State University, UNITED STATES

## Abstract

Downy mildew is caused by *Plasmopara viticola*, an obligate oomycete plant pathogen, a devasting disease of grapevine. To protect plants from the disease, complex III inhibitors are among the fungicides widely used. They specifically target the mitochondrial cytochrome *b* (cyt*b*) of the pathogen to block cellular respiration mechanisms. In the French vineyard, *P*. *viticola* has developed resistance against a first group of these fungicides, the Quinone outside Inhibitors (QoI), with a single amino acid substitution G143A in its cyt*b* mitochondrial sequence. The use of QoI was limited and another type of fungicide, the Quinone inside Inhibitors, targeting the same gene and highly effective against oomycetes, was used instead. Recently however, less sensitive *P*. *viticola* populations were detected after treatments with some inhibitors, in particular ametoctradin and cyazofamid. By isolating single-sporangia *P*. *viticola* strains resistant to these fungicides, we characterized new variants in the cyt*b* sequences associated with cyazofamid resistance: a point mutation (L201S) and more strikingly, two insertions (E203-DE-V204, E203-VE-V204). In parallel with the classical tools, pyrosequencing and qPCR, we then benchmarked short and long-reads NGS technologies (Ion Torrent, Illumina, Oxford Nanopore Technologies) to sequence the complete cyt*b* with a view to detecting and assessing the proportion of resistant variants of *P*. *viticola* at the scale of a field population. Eighteen populations collected from French vineyard fields in 2020 were analysed: 12 showed a variable proportion of G143A, 11 of E203-DE-V204 and 7 populations of the S34L variant that confers resistance to ametoctradin. Interestingly, the long reads were able to identify variants, including SNPs, with confidence and to detect a small proportion of *P*. *viticola* with multiple variants along the same cyt*b* sequence. Overall, NGS appears to be a promising method for assessing fungicide resistance of pathogens linked to cyt*b* modifications at the field population level. This approach could rapidly become a robust decision support tool for resistance management in the future.

## Introduction

Diseases caused by fungal plant pathogens can damage plant and crops and have a very destructive impact on agriculture activities and food production. *Plasmopara viticola* is an obligate oomycete plant pathogen and the causal agent of downy mildew, the most devastating disease of grapevine. *P*. *viticola* infects all green organs of host plant and has an alternating life cycle between sexual overwintering phase and asexual multiplication during the growing season, causing primary and secondary infection cycles respectively [1–6]. Currently, downy mildew management strategies include multi-site fungicides such as copper-based fungicides, e.g. Bordeaux mixture and dithiocarbamates, as preventive treatment. Subsequently, single-site fungicides—such as phenylamides (e.g. metalaxyl), mitochondrial complex III Qo site inhibitors (QoIs; e.g. azoxystrobin), carboxylic acid amides (CAA; e.g. mandipropamid), and more recently, complex III Qi site inhibitors (QiIs; e.g cyazofamid and amisulbrom)—are often introduced into management programmes (see Fungicide Resistance Action Committee–FRAC—Code List 2021 for more details [7]). Complex III inhibitors target the mitochondrial cytochrome *b* (cyt*b*) protein and block cellular respiration mechanisms [8].

The mitochondrial respiratory chain consists of multifunctional, oligomeric membrane enzyme complexes. Cytochrome *bc1* complex (complex III) is a key enzyme in the mitochondrial electron transport chain. Cyt*b* is a subunit of complex III that catalyses the transfer of electrons from ubiquinol to cytochrome *c*, resulting in protons translocation and energy transduction. The cyt*b* protein contains eight transmembrane helices encoded by the cyt*b* gene. Widely used complex III inhibitors target sites on these helices with different strategies [8, 9].

Fungicides known as QoI’s block mitochondrial respiration by binding to the Qo site [9, 10]. Isolates resistant to QoI fungicides were detected in field populations of many plant pathogens such as *Erysiphe necator* [11, 12], *P*. *viticola* [13], *Alternaria sp*. [14, 15], *Mycosphaerella graminicola* [16, 17] and many others [7]. In French vineyard, populations of downy mildew (*P*. *viticola)* have developed resistance to QoI’s fungicides through the substitution of a single amino acid G143A in the cyt*b* [9, 13, 18, 19]. The use of this group of single-target fungicides was restricted after the development of widespread resistance.

Fungicides known as QiI’s inhibit the reduction of quinol in the Qi site near the mitochondrial matrix. These fungicides are very effective against oomycetes and play an important role in nowadays downy mildew management programmes. However recently, populations of *P*. *viticola* less sensitive to ametoctradin (QoSI fungicide, Quinone outside Stigmatellin binding sub-site Inhibitor; FRAC Code List 2021 [7]) and cyazofamid (QiI fungicide), have been detected in vineyards [20, 21]. The origin of this resistance was unknown and several hypotheses could be put forward. Specific resistance could result from changes in the *P*. *viticola* cyt*b* sequence already reported, such as S34L which is thought to destabilise fungicide binding in the case of ametoctradin [22], or it could be the result of new modifications never before described. Alternatively, or in combination, and although mutations in inhibitor binding sites within the cyt*b* account for most of the reported cases of resistance, resistance may arise from a second mechanism involving the overexpression of an alternative oxidase (AOX) when the cyt*b* is inhibited ([22] for review). This mechanism of alternative respiration pathway was already observed in resistant populations of *P*. *viticola* collected in France [20, 21]. Therefore, populations can be resistant without showing changes in their cyt*b* sequence.

In order to better characterize the origin of the new resistance observed in the vineyard, the objective of this study was to isolate ametoctradin- and cyazofamid-sensitive and -resistant single-sporangia strains by biological tests and to analyse the resistant strains by molecular approaches. A leaf disc sensitivity bioassay was performed on the isolated strains to investigate cross-resistance between the fungicides QoI, QiI and QoSI. The cyt*b* sequences of the single-sporangia strains were obtained by Sanger sequencing and analysed to characterize possible new molecular mechanisms of resistance to these fungicides in *P*. *viticola*, i.e. the occurrence of new mutations. Sanger sequencing is suitable in this case because the composition of the single-sporangia is homogeneous and contains only one type of DNA, however this method is not adapted in the case of a mixture of DNA when several strains are present. In an effort to go further and analyse cyt*b* polymorphism at a field population scale, NGS technologies were exploited. They have first been benchmarked to sequence the complete cyt*b* from single-sporangia strains and then applied to the analysis of *P*. *viticola* field populations. Still relatively uncommon in studies associated with fungicide resistance of plant pathogens and usually targeting full pathogen genomes [23, 24], short-read (Ion Torrent and Illumina) and long-read (Oxford Nanopore Technologies, ONT) technologies were tested and compared in our study. They were found to be efficient in identifying and monitoring cyt*b* variants in field populations but also promising to become a robust decision support tool for fungicide resistance management, potentially in the near future, directly in the field.

## Materials and methods

### *P*. *viticola* populations and culture conditions

Downy mildew infected leaves were collected randomly in 2016, 2017, 2018 and 2020 in different vineyards from France wine regions (e.g. Bordeaux area, South-western region, Provence, Rhone Valley, Champagne …). Sampling was carried out in vineyards with fungicide protection programs including or not complex III inhibitors. More than 50 leaf discs surrounding infected lesion (oil spot) per sample were prepared from collected leaves. Leaf discs were placed onto Petri dish, washed with distilled water and dried at room temperature. After 24 h incubation, new downy mildew sporangia were collected with cotton swabs in sterile water to inoculate decontaminated and fungicide-free leaves of the grape cultivar *Vitis vinifera* cv. Cabernet-Sauvignon. Inoculated leaves were incubated in Petri dishes for 7 days at 22°C with a 16 hours of light/8 hours of dark photoperiodic lighting. Freshly produced sporangia were harvested to inoculate testing leaf discs.

### Chemical fungicides

Commercial formulations of ametoctradin (SNOOKER, concentrated solution containing 200 g active ingredient (AI) L^-1^, BASF, France) and cyazofamid (RANMAN TOP, concentrated suspension containing 160 g AI L^−1^, ISK Biosciences Europe, France) were tested. The fungicide formulations were dissolved in sterile distilled water. Stock solutions were stored at 4°C in the dark.

### Resistant single-sporangia isolates and cross-resistance assay

Leaf discs with sporulating colonies at 1 mg.L^-1^ of cyazofamid or ametoctradin with 100 mg.L^-1^ of SHAM (Salicylhydroxamic acid, Sigma-Aldrich, France), as inhibitor of alternative respiration (AOX), were used to isolate resistant strains of *P*. *viticola*. Collected populations with high level of non-specific resistance to the fungicide without SHAM were also included to isolate AOX-strains. Strains were isolated from a calibrated solution of oospores from each *P*. *viticola* population which theoretically contains 1 spore in 10μl (the volume of one droplet). The droplets were deposited on 100 discs (1 droplet per disc) and incubated until sporulation appeared. Single-sporangia sporulating spot was taken to inoculate new decontaminated leaves as described previously for further analysis.

Thus, monitoring of sensitivity assays using fungicide, alone or with SHAM, was used to select strains developing resistance involving either AOX respiration (non specific resistance) or cyt*b* target mutation mechanism. On the one hand, strains showing sporulation on leaf discs with both fungicide and SHAM had specific resistance mechanism, probably inducing cyt*b* target modification. On the other hand, strains growing only on fungicide without SHAM involve AOX activity.

To investigate cross-resistance, the *in vitro* sensitivity of these strains to ametoctradin and cyazofamid, applied alone or mixed with SHAM, was measured with increasing concentrations of each fungicide (0.01 mg.L^-1^ to 100 mg.L^-1^) mixed with spore solution at 1 x 10^+4^ spores mL-^1^. Ten discs were analysed for each condition (3 x 10 μl droplets deposited on one disc) and assays for each isolate were repeated three times per fungicide concentration. After 7 days, individual leaf discs were evaluated for disease incidence and sporulation using a rating scale based on visual observation of each droplet spot as described by Colcol and Baudoin [25].

### Total DNA extraction and PCR amplification

Single-sporangia strains of *P*. *viticola* growing leaf discs 7 days post inoculation were used as starting material for DNA extraction and PCR amplification. Total DNA was extracted using the Nucleospin® plant II kit (Macherey-Nagel GmbH & Co., Germany) according to the manufacturer’s recommendations. The same approach was followed for the field collected populations, except that DNA was extracted from freshly inoculated leaves as previously described.

PCR amplification of 1kb fragment of cyt*b* gene was carried out in 25 μL reaction mixtures with 30 ng of total genomic DNA, set of forward (5’-TGAACCTGTAAATTTAGCACAACAA-3’) and reverse (5’-ACAGGACATTGACCAACCCA-3’) primers (0.3 μM) and 1X premixed Phusion Flash High-Fidelity PCR Master Mix (Thermo Scientific, France). Amplifications were carried out in a thermal cycler LifeEco (BIOER Technology, France) using the following PCR program: initial denaturation at 94°C for 1 min followed by 35 cycles at 95°C for 30 s, 60°C for 1 min, and 72°C for 1 min and a final extension at 72°C for 10 min. Amplification products were then subjected to direct Sanger sequencing, using the same primers as for PCR amplification (GATC Biotech, Germany). To analyse cyt*b* gene partial sequence and investigate point mutations in resistant strains, the new sequences were aligned with Jalview [26] against a *P*. *viticola* cyt*b* sequence used as reference (accession number DQ459459.1).

### Pyrosequencing assay design for cyt*b* alleles quantification

Pyrosequencing technology was used to investigate allele’s frequencies of modifications detected in cyt*b* genes amplified from different samples: isolated field strains (selected single-sporangia strains) and field populations samples (mixed sporangia collected in the field). PCR reactions for pyrosequencing were performed in a final volume of 50 μl containing 30 ng of extracted DNA, 12.5 μl of PyroMark PCR Master Mix (Qiagen, France), 2.5 μl of CoralLoad concentrate (Qiagen, France), 5 μM of CONIPHY designed reverse and forward primers (*P*. *viticola* PyroID kit, CONIPHY, France) using PyroMark Assay Design version 2.0 (Qiagen, France). Target region of cyt*b* gene with the insertion of nucleotides was amplified using the following program: 95°C for 15 min, followed by 45 cycles (94°C for 30 s, 60°C for 30 s and 72°C for 30 s) and a final DNA extension at 72°C for 10 min (following manufacturer recommendations). PCR products (3 μl of the initial reaction mixture) were separated on a 1.2% agarose gel stained with SYBR^®^ Safe (Invitrogen™, France). Gel production and electrophoresis were conducted using TAE buffer (40 mM Tris-acetate pH 8.0, 1 mM EDTA). Pyrosequencing reactions were performed in a PyroMark Q48 Autoprep instrument (Qiagen, France) using PyroMark^®^ Q48 Advanced Reagents kit (Qiagen) with 3 μl of PyroMark Q48 magnetic beads (Qiagen) and 10 μl of biotinylated PCR products following manufacturer’s instructions. The sequencing primers provided in dedicated PyroID kit (CONIPHY, France) were used to detect E203-DE-V204 and L201S or E203-VE-V204. For validation of assays, 5 replicates of total DNA extracted from sensitive and resistant single sporangia isolates were analysed. Allele frequencies were estimated using PyroMark software.

### Cyt*b* PCR amplification and sequencing by short and long reads NGS technologies

NGS technologies were explored to characterize cyt*b* variants from sensitive or resistant single-sporangia isolated strains (7 DNA extracts tested). After this validation step, NGS were used to detect the presence and proportion of sensitive strains among the field populations collected in French vineyards in 2020 (18 DNA extracts tested). This was done by amplifying the complete cyt*b* from the extracted total DNA and sequencing the amplicons by short and long-read technologies.

For all the 25 samples analysed, complete cyt*b* was amplified using two different strategies: in 5 overlapping fragments (302 to 375 bp long) to perform short-read sequencing (Illumina or Ion Torrent), and in a single fragment (1457 bp long) to perform long-read sequencing (ONT).

Five primer pairs were designed by Ion AmpliSeq Designer (ThermoFisher Scientific, USA) using DQ459459.1 as a reference. For short-read approaches, the five overlapping fragments were amplified separately in 25 μl in a mix including 2X TaqMan™ Environmental Master Mix 2.0 (Applied Biosystems, USA), 1 μl of each primer at 10 nM and 10ng of genomic DNA. PCR amplifications were carried out in a Veriti Thermocycler (ThermoFisher Scientific, USA) using the following PCR conditions: initial denaturation at 94°C for 5 s followed by 35 cycles at 94°C for 30 s, 55°C to 65°C (depending on primers set) for 45 s, and 72°C for 1 min and a final extension at 72°C for 7 min. After purification and quantification, the five amplicons were then pooled for each sample in an equimolar manner.

For long-read approach, long-range PCR was performed to amplify the complete gene with the same mix composition as above except that the LongAmp HotStart Taq 2X Master Mix (New England Biolabs, France) was used instead. The primers used correspond to the most extreme forward and most extreme reverse primers designed above. The PCR program was the following: initial denaturation at 94°C for 10 min followed by 40 cycles at 94°C for 30 s, 60°C for 45 s, and 65°C for 2 min and a final extension at 65°C for 7 min.

For Ion Torrent, the barcoded libraries were built from pooled amplicons by a ligation protocol using the Ion Xpress™ Plus Fragment Library Kit and following the recommendations of the manufacturer. Pooled libraries were sequenced in SE 400 bp on a 318 Ion chip using a PGM sequencer (ThermoFisher Scientific, USA). For Illumina and ONT, fusion primers (i.e. containing partial adapters sequences specific of respectively Illumina or ONT) were used for the first PCR. In both cases, a second PCR was performed to add indexes/barcodes and to complete the libraries construction. Illumina barcoded libraries were sequenced with a Reagent kit v2 in PE 2x250 bp on a MiSeq sequencer. Nanopore libraries were finalized and barcoded with the PCR Barcoding kit protocol (SQK-PBK004, ONT) and sequenced on Flongle flow cells (FLO-FLG001, ONT) with Mk1C or MinION coupled with MinIT. High accuracy basecalling was performed for all Nanopore runs with Guppy version 3.2.9 or 4.3.4.

Short and long raw reads datasets were deposited to SRA database under BioProject accession PRJNA892502.

### NGS data analyses

Ion Torrent reads were analysed using the AmpliSeq design above and DQ459459.1 as a reference. Torrent Variant Caller (TVC) plugin proposed by ThermoFisher Scientific in the Torrent Suite Software was launched with a generic configuration for somatic and low stringency parameters. In this configuration, 2000 reads are considered to characterize new variants and low frequency detection is optimized (usually detection is not given below 5%).

Illumina reads were analysed with Galaxy Europe (**https://usegalaxy.eu**) or with our own instance. Roughly, for each sample, R1 and R2 reads were paired and then sorted by type of fragment. Five thousand reads were sampled for each of the 5 fragments and mapped with minimap2 [27] on the DQ459459.1 reference. The Bayesian genetic variant detector FreeBayes [28] was used (option simple diploid calling with filtering and coverage) to find polymorphisms and VCFlib [29] used to extract the variable positions in a table format with reads count.

Nanopore reads were analysed with a dedicated pipeline designed in command lines. Only reads higher than Q7 were considered. Five thousand reads were sampled for each sample and mapped on the DQ459459.1 reference with minimap2. Six regions or positions identified by this study as variable were searched with either a BLAST [30] approach or a Nanopolish (https://github.com/jts/nanopolish) analysis. Frequency at each position was estimated by counting the number of reads with the variant versus the total reads number.

To identify complete cyt*b* sequence with multiple polymorphisms, each single polymorphism was searched independently among the previous 5000 long reads sampled. All unique reads containing at least one polymorphism were then considered as a whole and proportion of reads containing one or multiple variants along the same read were computed. R package “ggvenn” was used to draw Venn diagrams and to obtain percentage of reads corresponding to each possible combination. Presence of several polymorphisms along the same read was checked by aligning read against the reference and confirmed by eye on randomly picked reads.

## Results and discussion

### Sensitivity of field isolates to ametoctradin and cyazofamid

Numerous field populations of downy mildew with low sensitivity to ametoctradin and cyazofamid at different levels have been observed in French vineyards since 2016. Resistant and sensitive populations were selected to generate single-sporangia isolates. More than 70 single-sporangia strains were isolated including 41 resistant to cyazofamid (QiI), 5 to ametoctradin (QoSI), and 3 strains showing non-specific resistance with AOX expression. Further investigations were undertaken on about 1/3 of them. Additional cross resistance studies with dose range assays and genotypic characterization were thus carried out on the panel of 22 single-sporangia strains listed in Table 1.

**Table 1:** MIC (Minimum Inhibitory Concentration) values for ametoctradin and cyazofamid observed for single-sporangia strains isolated from field-collected *P*. *viticola* populations and associated mutations or insertions detected in their cyt*b* gene compared to the reference DQ459459.1.

*P*.* viticola*	Ametoctradin (QoSI)			Cyazofamid (QiI)			Modification of cyt*b* sequence observed (Sanger)				
strains	Alone	+SHAM		Alone	+SHAM		G143A	S34L	E203-DE-V204	E203-VE-V204	L201S
CONI-01	0.3	<0.1	S	<0.1	<0.1	S	-	-	-	-	-
CONI-04	>100	0.3	AOX-R	30	<0.1	AOX-R	-	-	-	-	-
CONI-02	0.3	0.3	S	<0.1	<0.1	S	X	-	-	-	-
CONI-03	0.3	0.3	S	<0.1	<0.1	S	X	-	-	-	-
CONI-11	>100	0.3	S	30	<0.1	S	X	-	-	-	-
CONI-12	>100	0.3	S	30	<0.1	S	X	-	-	-	-
CONI-20	>100	100	**R**	<0.1	<0.1	S	-	X	-	-	-
CONI-22	>100	100	**R**	<0.1	<0.1	S	-	X	-	-	-
CONI-06	>100	100	**R**	<0.1	<0.1	S	X	X	-	-	-
CONI-13	100	100	**R**	<0.1	<0.1	S	X	X	-	-	-
CONI-05	1	1	S	100	30	**R**	-	-	X	-	-
CONI-07	1	0.3	S	100	30	**R**	-	-	X	-	-
CONI-15	1	0.3	S	100	30	**R**	-	-	X	-	-
CONI-16	1	0.3	S	100	30	**R**	-	-	X	-	-
CONI-08	>100	0.3	S	100	30	**R**	-	-	X	-	-
CONI-09	>100	0.3	S	100	30	**R**	-	-	X	-	-
CONI-10	>100	0.3	S	100	30	**R**	-	-	X	-	-
CONI-17	1	0.3	S	100	30	**R**	-	-	X	-	-
CONI-31	>100	0.3	S	30	30	**R**	-	-	-	-	X
CONI-38	10	0.3	S	30	30	**R**	-	-	-	-	X
CONI-39	>100	0.3	S	30	10	**R**	-	-	-	X	-
CONI-41	0.3	0.3	S	30	10	**R**	-	-	-	X	-

A dash indicates no modification, a cross indicates detection of the variant. AOX-R: AOX resistant; R: resistant; S: Sensitive, indicates resistance state when AOX mechanism is blocked by SHAM and where resistance is likely to result mainly from changes in cyt*b*.

### Molecular analysis of field isolates *P*. *viticola* cyt*b* gene

#### Confirmation of S34L substitution in cytochrome *b* conferring resistance to ametoctradin

**Fig 1:**
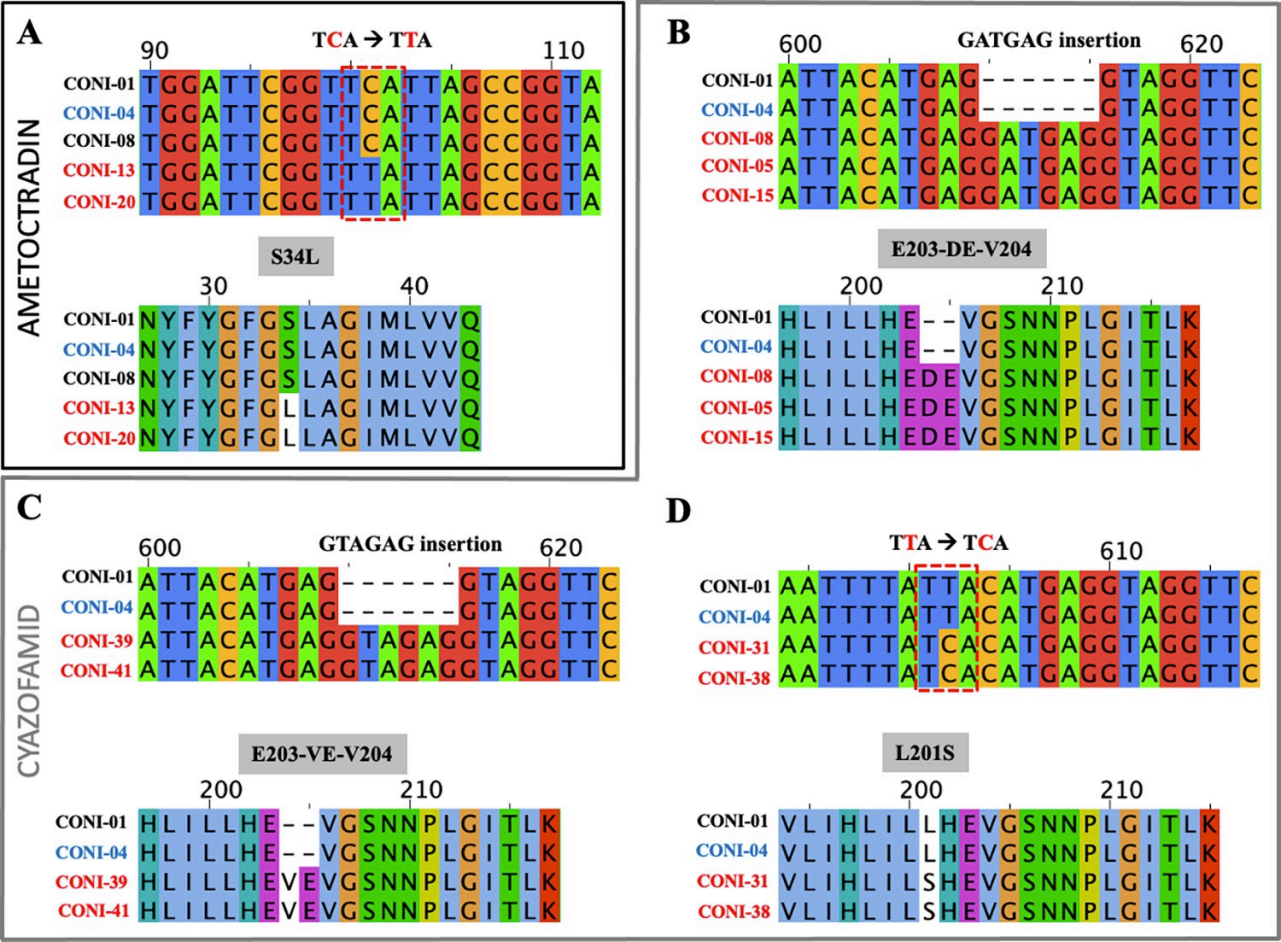
Alignment of cyt*b* partial sequences of cyazofamid and ametoctradin *P*. *viticola* resistant isolates. In black: sequence of sensitive strain. In red: sequences of strains resistant to cyazofamid or ametoctradin accordingly. In blue: AOX strain. The cyt*b* variations observed for the resistant isolates are non-synonymous changes or multiple 3-base insertions all leading to amino acid changes in the protein. Sequences of strains with S34L substitution (A), GATGAG insertion (B), GTAGAG insertion (C) and L201S substitution (D).

Of the 12 strains of *P*. *viticola* showing inhibited growth at 100 mg.L^-1^ of ametoctradin when applied alone, only four isolates retained this inhibition when the fungicide was mixed with SHAM (CONI-06, CONI-13, CONI-20, CONI-22; Table 1). These strains exhibited a high level of resistance (Resistance Factor = 1000) to ametoctradin compared to the sensitive strain CONI-01 (MIC <0.1 mg.L^-1^). These strains are sensitive to cyazofamid (<0.1 mg.L^-1^) applied alone or with SHAM (Table 1). The loss of sensitivity of these strains appears to be caused by a change in the target specifically affecting the mode of action of ametoctradin. Analysis of the cyt*b* gene sequences of these strains, obtained by Sanger sequencing, reveals the presence of a single nucleotide mutation switching cytosine to thymine at position 101 (TCA → TTA). This mutation results in the substitution of the amino acid serine by a leucine at position 34 (S34L) in ametoctradin resistant *P*. *viticola* isolates (Fig 1A). No cross resistance was identified in strains carrying S34L substitution with cyazofamid. Among the strains carrying the G143A substitution, conferring resistance to QoI fungicide, the cyt*b* gene sequence of two isolates (CONI-06 and CONI-13) contained both the S34L and G143A substitutions (Table 1). When tested in leaf discs bioassays, these strains are resistant to both pyraclostrobin (QoI) and ametoctradin. Ametoctradin was first described as a QoSI inhibitor acting on the Qo site [31]. However, the S34 serine of *P*. *viticola* cyt*b* is located in the Quinone inside site (Qi) suggesting that ametoctradin inhibits mitochondrial respiration by interacting with complex III in the Qi site. The results suggest that the mode of action of ametoctradin in the Qi site is different from that of cyazofamid. They confirm recent research on the mode of binding of ametoctradin to its target site in the cytochrome *bc1* complex, showing that ametoctradin is able to interact with both Qo and Qi sites [20, 22, 32].

#### L201S substitution and two different 6 bp insertions (E203-DE-V204 and E203-VE-V204) in cyt*b* coding gene confer resistance to cyazofamid

Sensitivity bioassays revealed that 41 isolates of *P*. *viticola* were resistant to cyazofamid applied alone or mixed with SHAM at a discriminating dose (1mg.L^-1^). Further analysis of 12 strains showed that the development of *P*. *viticola* in 10 isolates was inhibited at 30 mg.L^-1^ and in 2 isolates (CONI-39 and CONI-41) at 10 mg.L^-1^ of cyazofamid+SHAM compared to the sensitive strain CONI-01 (<0.1 mg.L^-1^) (Table 1). The cyt*b* sequences of 31 of these isolated resistant strains show an insertion of 6 nucleotides, GATGAG, compared to the cyazofamid-sensitive strains and the reference DQ459459.1. This short sequence insertion results in an amino acid sequence change with two additional amino acids (E203-DE-V204) (Fig 1B). A second insertion of 6 nucleotides, GTAGAG, is observed in two other strains causing another change in amino acid E203-VE-V204 (Fig 1C). Finally, the cyt*b* sequences of 2 isolated strains, referenced CONI-31 and CONI-38, inhibited with 30 mg.L^-1^ of cyazofamid+SHAM, contain a single nucleotide mutation at position 602 (TTA→TCA) inducing an amino acid substitution, L201S, in cyt*b* protein (Fig 1D). These three variants, all located in the same short region of the cyt*b* gene, confer resistance to cyazofamid but no cross resistance to ametoctradin. To our knowledge, fungicide resistance caused by target modification has only occurred with single nucleotide polymorphism (SNP) in almost all described plant pathogens confronted with fungicides with a single-site mode of action [7, 33]. Inserting a short sequence into the gene encoding the fungicide target, and probably changing the conformation of the protein, without altering the function of the protein, could be a new way for the pathogen to bypass the action of the fungicide. Interestingly, a similar amino acid modification was introduced into the yeast cyt*b* mutant model and no effect was observed on yeast growth [34]. Comparative protein structure modelling suggests that the insertion of two amino acid E203-DE-V204 interferes with cyazofamid binding to cyt*b* in *P*. *viticola* [34]. The description of the six sequence variants in the *P*. *viticola* cyt*b* gene that are associated with resistance to cyazofamid, ametoctradin or QoI fungicides, identified in this study or from the literature, is presented in Table 2. In the remainder of this study, we looked specifically for these 6 variants.

**Table 2:** Description of *P*. *viticola* variants of cyt*b* gene and associated resistance to cyazofamid, ametoctradin or QoI fungicides considered in this study.

Variants	AA modifications	Reference	Variant	Type	Resistance	References
Variant 1	E203-DE-V204	-	GATGAG	Insertion (6 bp)	Cyazofamid	[20], this study
					QiI fungicide	
Variant 2	E203-VE-V204	-	GTAGAG	Insertion (6 bp)	Cyazofamid	This study
					QiI fungicide	
Variant 3	L201S	T	C	Substitution	Cyazofamid	This study
					QiI fungicide	
Variant 4	S34L	C	T	Substitution	Ametoctradin	[20, 21], this study
					QoSI fungicide	

Variant 5	F129L	T	A/G/C	Substitution	QoI fungicides	FRAC 2021
Variant 6	G143A	G	C	Substitution	QoI fungicides	FRAC 2021, this study

DQ459459.1 is used as the reference sequence.

#### Pyrosequencing quantification of L201S substitution and the two insertions in *P*. *viticola* cyt*b* gene

Two pyrosequencing-based methods were developed to quantify the frequency of the 3 variants in the samples. The frequencies of E203-DE-V204 and L201S were measured simultaneously in a first assay (S1 Fig). The frequency of E203-VE-V204 was evaluated in a second assay (S2 Fig). Pyrosequencing experiments were validated on DNA extracted from cyazofamid-resistant single-sporangia isolates. They showed that cyazofamid-resistant isolates had between 96 to 98% insertions E203-DE-V204, E203-VE-V204 or substitution L201S, which corroborates the Sanger analysis (Fig 1) and the sensitivity tests (Table 1). Finally, to estimate the background noise, the two pyrosequencing methods were applied to the sensitive wild-type strain (CONI-01) that present no sequence variants, and to plasmid constructs in which partial sequences of sensitive cyt*b* have been inserted (not shown). In all cases, low frequencies (2–8%) of variants were observed (S1 and S2 Figs) allowing to fix a threshold for accurate detection. For further analyses, only populations with a variant frequency above 10% were considered as significantly detected by pyrosequencing.

When applied to DNA extracted from field-collected populations, only the E203-DE-V204 variant was detected at different proportions by pyrosequencing analyses. Frequencies of the E203-DE-V204 variant below 10%, or close to background noise, were measured in five cyazofamid-sensitive populations (100% efficacy; Table 3). Eleven field populations of *P*. *viticola* harboured the E203-DE-V204 variant with frequencies ranging from 10 to 96%. Both observations, on single sporangia isolates and field populations, show a significant correlation between genetic profiling by pyrosequencing and phenotypic characterization with *in vitro* bioassays. The E203-VE-V204 and L201S variants were not detected in the field populations tested (Table 3), suggesting that the E203-DE-V204 insertion may be predominant in the field and probably at the origin of cyazofamid-resistant downy mildew populations in French vineyards.

**Table 3:** Characterization of fungicide resistance of 18 field populations of *P*. *viticola* collected in French vineyards in 2020 (S3 Fig for map location).

Samples		Biological test on leaf discs (% efficacy)		Cyt*b* allele frequency quantification								
Field population	Location French department	Ametoctradin (1mg/l) + SHAM (100mg/l)	Cyazofamid (1mg/l) + SHAM (100mg/l)	qPCR		Pyrosequencing	NGS sequencing					
							FreeBayes / Illumina (Short reads)			BLAST / ONT (Long reads)		
				% G143A	% S34L	% E203-DE-V204	% G143A	% S34L	% E203-DE-V204	% G143A	% S34L	% E203-DE-V204
CONI-P1	33	100	35	-	-	52 ± 0.7	-	-	51	-	-	48
CONI-P2	30	100	100	-	-	3 ± 0.4	-	-	-	-	-	-
CONI-P3	47	100	100	-	-	4 ± 1.3	-	-	-	-	-	-
CONI-P4	13	100	100	-	-	4 ± 1.6	-	-	-	-	-	-
CONI-P5	51	98	0	8	-	81 ± 1.1	8	-	81	8	7	81
CONI-P6	51	Not tested	97	94	-	9 ± 0.4	93	-	-	94	-	6
CONI-P7	51	Not tested	82	63	-	33 ± 0.9	60	-	30	64	-	28
CONI-P8	51	Not tested	60	46	-	31 ± 0.7	43	-	30	46	-	28
CONI-P9	51	Not tested	23	-	-	96 ± 1.1	-	-	95	-	-	95
CONI-P10	17	100	69	59	-	37 ± 1.1	55	-	35	57	-	36
CONI-P11	33	100	85	-	-	25 ± 1.1	13	-	23	13	-	22
CONI-P12	33	100	100	56	-	6 ± 0.9	47	-	-	48	-	-
CONI-P13	17	47	67	73	88	10 ± 0.9	69	94	5	70	93	5
CONI-P14	17	40	100	74	79	4 ± 1.3	82	91	-	85	92	-
CONI-P15	32	65	85	-	-	7 ± 0.9	6	9	-	6	12	-
CONI-P16	33	55	57	-	18	85 ± 2.4	-	10	85	-	14	84
CONI-P17	33	52	88	12	49	15 ± 1.1	8	57	12	9	59	12
CONI-P18	51	57	80	39	63	13 ± 2.9	43	64	-	43	64	-

Biological tests and associated proportions of *P*. *viticola* sequence variants observed from cyt*b* gene estimated by qPCR, pyrosequencing and NGS sequencing are given. For the biological tests, value of 100% indicates no resistance measured in the population for the fungicide, and 0% means totally resistant. Sequence cyt*b* variants considered are described in Table 2. Only E203-DE-V204, S34L and G143A variants were detected in the field populations analysed and are given. All values are expressed in %. A dash indicates non detected or value below limit detection (threshold of 5% considered for NGS).

Molecular characterisation tools, including quantitative PCR (qPCR), are widely used in the detection and quantification of fungicide resistance in plant pathogens [11, 21, 35–39]. However, in this particular case, all of the qPCR assays we set up to analyse the two six-nucleotides insertions that were found in the *P*. *viticola* cyt*b* gene failed. Therefore, the detection of the two insertions relies solely on the pyrosequencing approach. Allele quantification using pyrosequencing technology has been used for SNP detection and quantification of resistance to QoI and CAA fungicides in *P*. *viticola* [40, 41]. On the other hand, the target site modification related to succinate dehydrogenase inhibitors (SDHI) resistance in *Pyrenophora teres* was identified using pyrosequencing [42]. Field monitoring of the G143A substitution in *Cercospora sojina* [43] or DMI (DeMethylation Inhibitors) and SDHI resistance in *Ramularia collo‑cygni* populations [44] were investigated by pyrosequencing. The pyrosequencing method is a powerful alternative method allowing detection and quantification of single nucleotide polymorphism (SNP) but also, as shown in this study, enabling detection of insertion or deletion of nucleotides to ascertain gene modification in fungicide resistance. Pyrosequencing and qPCR methods are accurate and less time-consuming approaches than biological tests. However, these methods are only relevant for fungicide resistance mechanisms where target changes are known [39]. In addition, several reactions may be required for a single sample if various mutations associated with fungicide resistance are to be assessed, such as G143A, S34L and L201S for example.

### NGS analyses of cyt*b* genes from field *P*. *viticola* isolates

Considering the limits of the two previous methods, we explored other approaches to identify new variants or to assess the presence of known cyt*b* variants and their proportion at a population level. In this context, the more recent development of new sequencing technologies, such as short- and long-reads NGS, is promising and opens up opportunities to improve the quality and sensitivity of molecular detection of pathogen resistance to fungicides. For example, Whole Genome Sequencing (WGS) is already widely used to monitor antimicrobial resistance [45], sometimes combining short- and long-reads approaches [46]. In medical applications, ONT long-reads generated in real-time offer the promise of very rapid diagnosis to identify pathogens and antibiotic resistance [47]. ONT is proving to be an interesting tool for plant monitoring to detect plant viruses on a routine basis [48]. Finally, large-scale genomic studies on plant disease resistance have been possible with these high-throughput and cost-effective tools to clarify the interactions between legumes and pathogens [49]. At the gene level, only a few studies have already reported successful cyt*b* sequencing using short-read NGS method to study pathogen resistance to fungicides. This was the case, for example, for detecting and characterizing resistance of the wheat pathogen (*Zymoseptoria tritici*) to QoI [50]. However, NGS technologies have been little explored in the literature for this specific purpose. We therefore decided to test short- and long-reads sequencing with the aim of developing new methods to obtain a more efficient and rapid characterization of new or known cyt*b* variants of *P*. *viticola* in the context of fungicide resistance and which would be suitable for large-scale population monitoring.

#### Benchmarking of the NGS technologies to accurately detect variants from single-sporangia strains

The complete cyt*b* gene amplified from DNA extracts of 7 single-sporangia strains was analysed by short- and long-reads sequencing to investigate the reliability of detection of the 3 cyt*b* variants we described with Sanger (Fig 1, Table 2). The wild-type strain CONI-01 is sensitive and served as a control, the other strains were characterized as resistant and display either the E203-DE-V204, E203-VE-V204 or L201S variant. Both the short-read (Ion Torrent) and long-read (ONT) approaches detected the expected variant at a high frequency (all values > 92% and up to 100%) in the expected samples (S1 Table). The TVC tool used for the Ion Torrent reads, which only reports variants with a frequency greater than 5%, was successful in finding the specific variant with high values for quality detection. As the E203-VE-V204 variant is a 6 bp duplicate of the original sequence that is inserted, two alternative mappings are possible (Fig 1). Few mapping artefacts were therefore observed which could explain the slightly lower frequency detected by TVC for this variant (92%). For the long ONT reads, both pipelines (BLAST and Nanopolish) gave close, similar and accurate estimates of the expected variant (>95%). Low frequencies (below 2% for BLAST and <5% for Nanopolish) are however detected in almost all samples where variants were not expected. This suggests that below this threshold, detection of a variant cannot be trusted. According to this preliminary analysis, we arbitrarily set the variant detection threshold for all NGS methods (short or long reads) at 5% and this can be considered as a limit of the method’s sensitivity. Both the short-read (Ion Torrent) and long-read (ONT) approaches succeeded in detecting the expected variant at a high frequency (all values > 92% and up to 100%) for the single-sporangia samples It should be noted that the observation of a low percentage of the unexpected variant in the single-sporangia strains could be explained in different ways: by the experimental error rate (errors induced during PCR amplification or related to the sequencing step), by the sensitivity of the tools used to analyse the reads, but could also be an indication of heteroplasmy. Heteroplasmy is a widespread, but relatively rare, phenomenon observed in many species, consisting in the presence of different types of mitochondrial DNA in the same cell [21]. Heteroplasmy has been associated with a possible means for some species to rapidly adapt to fungicides and develop resistance [51, 52]. In particular, heteroplasmy has been reported for the G143A variant associated with QoI resistance [19, 53], including for *P*. *viticola* [21]. However, in our case, the low frequencies detected for the variants in sensitive strains are more likely a consequence of the background of the method than of heteroplasmy.

#### Exploiting NGS technologies to detect proportion of variants in *P*. *viticola* field populations

DNA extracted from 18 field populations of *P*. *viticola* collected in 2020 from French vineyards (S3 Fig) was used to amplify the complete cyt*b* into short fragments for Illumina sequencing or into a single long fragment for ONT sequencing. Both sequencings revealed striking similar results in the detection and proportion of each variant among the samples, with high correlation values and R^2^ > to 99% (Table 3). Two small differences were observed with the detection by the ONT approach of two variants in two additional samples (the S34L variant for CONI-P5 and the E203-DE-V204 variant for CONI-P6). However, both are present in a very low proportion (7% and 6% respectively, Table 3), close to the 5% threshold chosen for the detection of variants.

Of the 18 samples tested, three were sensitive to fungicides (ametoctradin and cyazofamid). In these sensitive samples, none of the six cyt*b* known variants (Table 2) were detected by NGS sequencing. The L201S, E203-VE-V204 and F129L variants were not apparent in any sample suggesting that they may be rare. However, the 3 other variants, E203-DE-V204, S34L and G143A were detected. The G143A variant, well known in the literature and first detected in French vineyard in 2003 [54] with several occurrence [13], is the most present, with detection in 12 samples. Then, the E203-DE-V204 variant (Table 2) is found in more than 50% of the samples screened in this study, with 11 samples featuring the modified cyt*b* in different proportions (5 to 95%). This insertion, which we first discovered in 2016 [20], is thus occuring in several populations collected 4 years later and at high frequencies (> to 25% in 7 samples; Table 3). However, these results should be taken with caution given the small number of samples analysed and need to be confirmed in a larger scale study. Finally, the S34L variant was identified in 6/7 samples, in varying proportions (7 to 94%). The same variant was previously reported in 2017 in 7 of 33 vineyards tested using allele-specific PCR assays [21].

The results observed for NGS sequencing (short- or long-read) are mostly in agreement with other molecular methods used independently to investigate the presence of the E203-DE-V204 variant by pyrosequencing, or the presence of G143A and S34L variants by qPCR. Nonetheless, NGS were found to be potentially more sensitive than qPCR in detecting low frequency variants in CONI-P11 and CONI-P15. The results are also in agreement with biological tests performed to evaluate resistance to ametoctradin (associated with the S34L variant) or to cyazofamid (associated with the E203-DE-V204 variant).

#### Long reads can detect combination of variants along the same cyt*b* sequence

Sequencing of short reads (Illumina or Ion Torrent) is attractive because of the high quality of the reads (reads with Quality Value (QV)>25) and the low error rate, which is even lower in Illumina reads than in Ion Torrent reads [55]. Both technologies are therefore ideal for identifying new substitutions or modifications with confidence. However, while short overlapping reads can be used and assembled to reconstruct a longer sequence when no diversity is present in the sample, they cannot be used to perform accurate phasing or to associate modifications occurring on different fragments in different proportions. On the contrary, long ONT reads have a lower quality (reads with QV<12), which sometimes makes it more difficult to identify low frequency substitutions. Still, the diversity within a population of long fragments can be confidently evaluated, allowing the clear identification of different haplotypes and their proportion on the long sequences.

**Fig 2:**
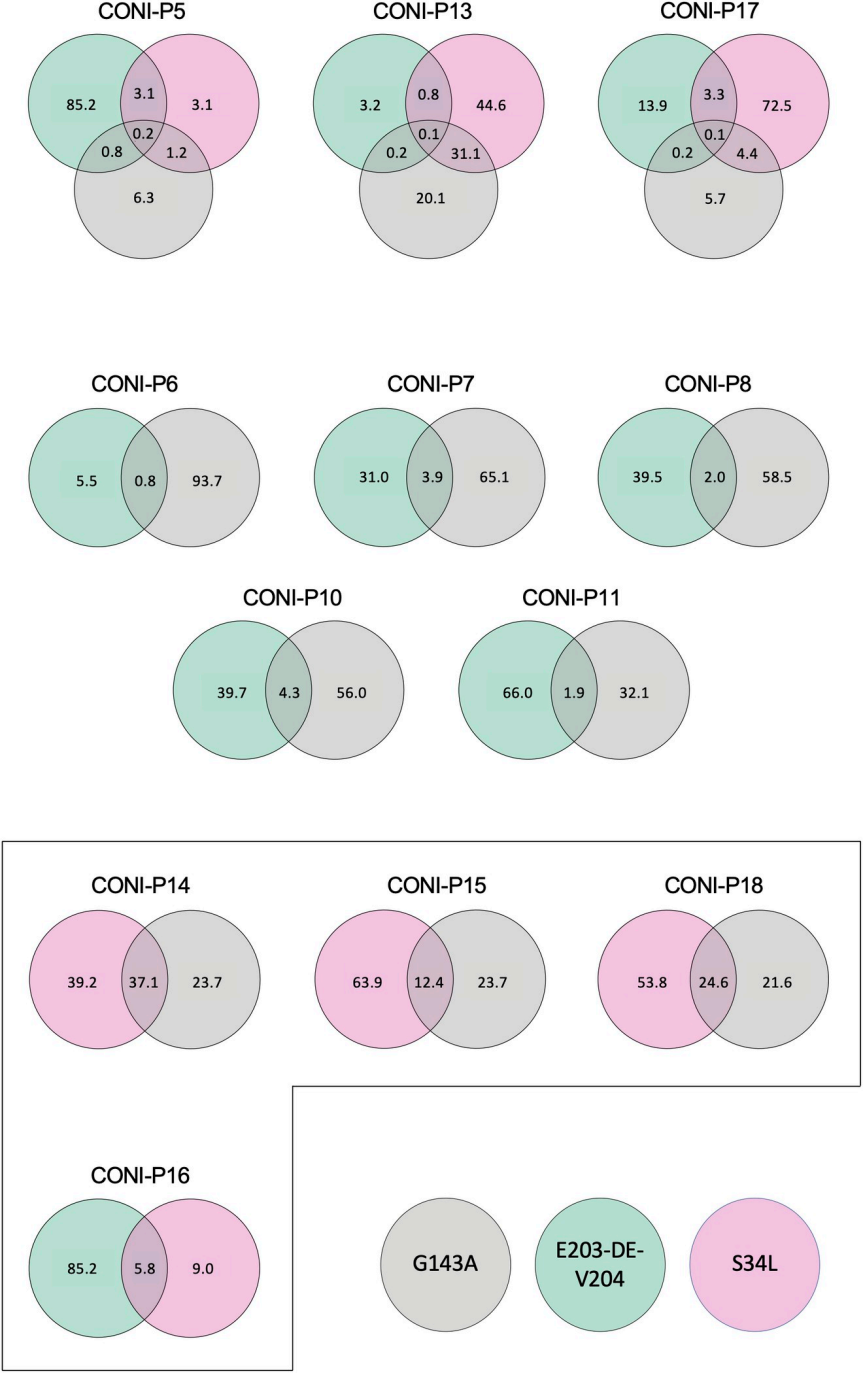
Cyt*b* sequences from field populations of *P*.*viticola* may show several changes correlated with resistance to cyazofamid and ametoctradin. For each population where at least two different variants were detected (Table 3), and considering only ONT reads with variants, the percentage of each type of variant (E203-DE-V204, S34L or G143A; Table 2) observed in the same read is indicated in the Venn diagram. Framed diagrams indicate populations where more than 5% of the reads have two different variants on the same sequence.

Taking advantage of these NGS specificities, we investigated in our datasets whether in some cases certain variants could be present in combination along the same cyt*b* sequence or whether each variant corresponds to different cyt*b* sequences. As the variants searched for in this study were amplified on different short fragments, only the long reads, i.e. complete cyt*b* sequences, were explored. The reads obtained for 12 populations with at least 2 types of variants were analysed in more detail (Table 3, Fig 2). However, although we detect the possible simultaneous presence of some variants (e.g. G143A and E203-DE-V204), a low frequency could simply be the result of the background noise of the method. For this reason, the same 5% threshold was applied to consider a proportion of reads with a combination of variants as significant, leading to the restriction of the dataset to 4 populations only.

In the CONI-P16 sample, approximately 40% of the reads identified with the S34L variant, also contained the E203-DE-V204 variant (Fig 2). Very few reads (5.8%) were affected by this combined pattern, suggesting that only a few strains of *P*. *viticola* may be double resistant to cyazofamid and ametoctradin at present, or that the simultaneous presence of both cyt*b* modifications may be deleterious to *P*. *viticola*. Consequently, the probability of isolating putatively viable single-sporangia strains by the experimental process described above was extremely low, and thus the possibility of describing them by Sanger sequencing alone was very limited. In contrast, the ONT approach used in this study illustrates the method’s ability to catch the appearance of multi-variant strains in a field population at an early stage by long-read sequencing, with deep sequencing coverage of the complete cyt*b* amplified by PCR.

When looking for the possible association of the E203-DE-V204 variant and the S34L variant with the G143A variant in the same cyt*b* sequence in all populations detecting at least two of them (Fig 2), one can observe that variant S34L and G143A variant are more often associated than any other combination. These two variants are SNPs that have been consistently described in the literature for years as key mutations in cyt*b* associated with resistance in plant pathogens [7]. For the 3 *P*. *viticola* populations collected that shared only S34L and G143A (CONI-P14, CONI-P15 and CONI-P18), 12.4% to 37.1% of the reads with one of these variants also carried the second variant. This also applies to CONI-P13 (31.2%), in which 3 different variants were observed. In 3 of these 4 populations, both variants reach high percentages (Table 3), and are only detected at low frequencies in CONI-P15. It should be noted that the 4 populations are located in different departments, in different regions of France, which suggests that this event may have occurred independently several times. The combination of G143A and S34L observed on long reads suggests the existence of possible viable strains resistant to multiple fungicides (QoI and ametoctradin), and may not be so rare. This is confirmed by the 2 resistant single-sporangia strains we were able to isolate in this study that combine both variants (CONI-06 and CONI-13; Table 1) and by a similar strain identified in a previous publication [21].

## Conclusions

This study provides an overview of resistance to complex III inhibitors in downy mildew populations in French vineyards. In addition to the well-known resistance of *P*. *viticola* to QoI fungicides, strains resistant to other complex III inhibitors, such as cyazofamid (QiI) and ametoctradin (QoSI), were detected. First, we identified the cyt*b* variants of *P*. *viticola* associated with this new resistance using single-sporangia strains, isolated after an extensive bioassay study on populations collected in the field since 2016. We observed that *P*. *viticola* is able to develop resistance to ametoctradin by target modification with the S34L substitution. We also detected a more surprising type of target modification in cyazofamid-resistant populations that had not been previously described. This new mechanism consists of two different six-nucleotides insertions in the cyt*b* gene in the Qi site, i.e. the insertion of two amino acids at the protein level, E203-DE-V204 and E203-VE-V204. Finally, we found that the L201S amino acid substitution also confers resistance to cyazofamid. No cross resistance was observed between cyazofamid and ametoctradin in the isolated strains.

In a second step, we combined classical approaches (biological test assays, pyrosequencing and qPCR) and new sequencing approaches (short- and long-read NGS) to monitor the 6 variants conferring fungicide resistance, but this time on a population level and no longer on single-sporangia isolates. This is a first study to simultaneously associate all these methods at this level. This allowed us to compile a comparative table of the advantages and disadvantages of the different strategies that can be deployed to characterise resistance phenomena in pathogens exposed to fungicides (Table 4). The multi-approach analysis of 18 field populations collected in 2020 shows that many complex III inhibitors resistant phenotypes with different genotypes can co-exist in the same population. The use of long-read technology, with the possibility of sequencing complete genes and thus identify the presence of multiple resistance variants simultaneously present, provides insight into the population structure of the pathogen in response to fungicides at the molecular level. The detection of low frequencies of multi-fungicide resistant strains in the emergence phase can help to design fungicide resistance management strategies, mainly for newly introduced molecules. However in order to give reliable recommendations, molecular analysis alone is not sufficient to decide on the resistance situation, other factors need to be studied, such as the fitness of mutant strains and the impact on disease control in the field.

**Table 4:** Comparison of tools used for characterising fungicide resistance mechanisms in pathogens.

	Biological tests (Bioassays)	qPCR	Pyrosequencing	Sanger sequencing	NGS short-reads (Illumina)	NGS long-reads (Nanopore)
**Type of resistance detected**	All types of resistance detected (AOX or molecular mechanism)	Molecular mechanism only Resistance induced by sequence modification(s) of fungicide targets				
**Advantages**	Direct evidence of resistance to fungicide by exposing spores to different doses of product Detect resistant phenotype/pattern independently of resistance mechanism Essential to characterize fungicide resistance induced by target modification	Suitable for single nucleotide modification Rapid (in hours), sensitive, robust, and reproducible Applicable for high-throughput routine testing Detection and quantification Cost effective	Suitable for multi-sites mutation and indel. Rapid, robust, and reproducible Applicable for high-throughput routine testing Detection and quantification	Allows to describe new sequence modifications Can be used to identify polymorphism on single DNA samples (pure isolate/strain) Detection Cost effective	Allows to describe new sequence modifications Can be used to identify polymorphism on single DNA or mixed DNA samples Detection and quantification High throughput sequencing: many samples can be combined High quality sequencing with very few errors	Allows to describe new sequence modifications Can be used to identify polymorphism on single DNA or mixed DNA samples Detection and quantification High throughput sequencing: many samples can be combined Long regions or complete genes can be targeted Allows to identify multiple modifications combined on a single molecule
**Limits**	Time-consuming: results in days/weeks Testing obligate parasites requires fresh plant material Qualitative information Specific protocol for each pathogen	Applicable only for known and characterized target modification Failed in the case of multi-sites mutation Failed when modification include deletion/insertion	Applicable only for known and characterized target modification Requires a PCR amplification step Only small regions can be targeted (<200pb)	Maximum of 1 kb regions can be targeted Low throughput Not suitable for field population Requires a PCR amplification step Requires sequence analysis	Only small regions can be targeted (<400bp) Requires costly equipment and bioinformatic analysis	Lowest quality of sequencing with more errors Requires bioinformatic adapted analysis Emerging technology

In particular, short-reads sequencing have proven to be very effective in detecting and characterising new variants, and all NGS technologies in catching various proportion of resistant strains in field populations. The conclusions reached, which are strikingly consistent between the different NGS approaches, are in most cases consistent with results generated by other molecular experiments using independent methods traditionally used for this purpose. Taken together, NGS approaches show promise for monitoring fungicide resistance related to cyt*b* modifications or detecting new putative resistant strains in field populations of *P*. *viticola*, with improved sensitivity and at a cost that will be reduced by pooling many samples at once. This should allow earlier detection of the emergence and spread of certain resistances in vineyards against some fungicides, more quickly, on a larger geographical scale and with a greater number of samples, avoiding the use of less effective chemicals.

Although the mechanisms of resistance to complex III inhibitors are complicated and multiple, when associated to cyt*b* sequence modification, long-read ONT sequencing should especially be considered as an interesting tool to assess pathogen resistance potentially directly in the field due to the portability and small size of ONT sequencers, as has been done to identify plant viruses [56–58]. As the sequence error rate is expected to decrease drastically for long reads in the near future, it should be possible to better detect and characterise new cyt*b* resistant variants in field populations more rapidly, even if they are present at low frequencies. If ONT could revolutionise the diagnosis and biomonitoring of downy mildew in the field in the future [59], long ONT reads, when coupled with relevant biological parameters of the pathogen, could also be a robust decision support tool for fungicide treatments in vineyards, and more generally, for all plants of agricultural interest where resistance is associated with a target-sequence change.

## Supporting information

S1 Fig Pyrograms showing allele quantification of E203-DE-V204 and L201S variants from sensitive and cyazofamid-resistant *P*. *viticola* strains. Pyrograms show DNA extract of single sporangia isolates CONI-01 (A), CONI-16 (B) and CONI-38 (C). Nucleotide positions from 11 to 24 represent the variable region to be analysed to detect insertion E203-DE-V204 (B). Nucleotide in position 28 concerns quantification of L201S substitution (C).
(PDF)

S2 Fig Pyrograms showing allele quantification of E203-VE-V204 variant from sensitive and cyazofamid-resistant *P*. *viticola* strains. Pyrograms show DNA extract of single sporangia isolates CONI-01 (A) and CONI-39 (B). Nucleotide positions 8 to 10 represent the variable region to be analysed to detect insertion E203-VE-V204 (B).
(PDF)

S3 Fig Locations of the 18 field samples of *P*. *viticola* studied. The French wine regions where the populations sampled come from are indicated. Samples are identified by the CONI-PX number used in Table 3. The map is designed by @comersis.com.
(PDF)

S1 Table Detection and frequency of E203-DE-V204, E203-VE-V204 and L201S variants observed with short-read (Ion Torrent) and long-read (ONT) sequencing of cytb gene amplified from single sporangia strains. For Ion Torrent data, variants were searched without a priori with Torrent Variant Caller (somatic and low stringency parameters) and only variants above 5% are reported. For ONT data, two dedicated pipelines using either BLAST or Nanopolish were tested. The 3 variants were specifically targeted in all samples.
(PDF)
