# Supplementary material for: New insights from short and long reads sequencing to explore cytochrome b variants in Plasmopara viticola populations collected from vineyards and related to resistance to complex III inhibitors
Source: PLoS One. 2023 Jan 19;18(1):e0268385. doi: 10.1371/journal.pone.0268385 (PMC9851517; doi:10.1371/journal.pone.0268385)

Fig S1. Pyrograms showing allele quantification of E203-DE-V204 and L201S variants from sensitive and cyazofamid-resistant *P. viticola* strains. Pyrograms show DNA extract of single sporangia isolates CONI-01 (A), CONI-16 (B) and CONI-38 (C). Nucleotides position from 11 to 24 represents the variable region to be analysed to detect insertion E203-DE-V204 (B). Nucleotide in position 28 concern quantification of L201S substitution (C).

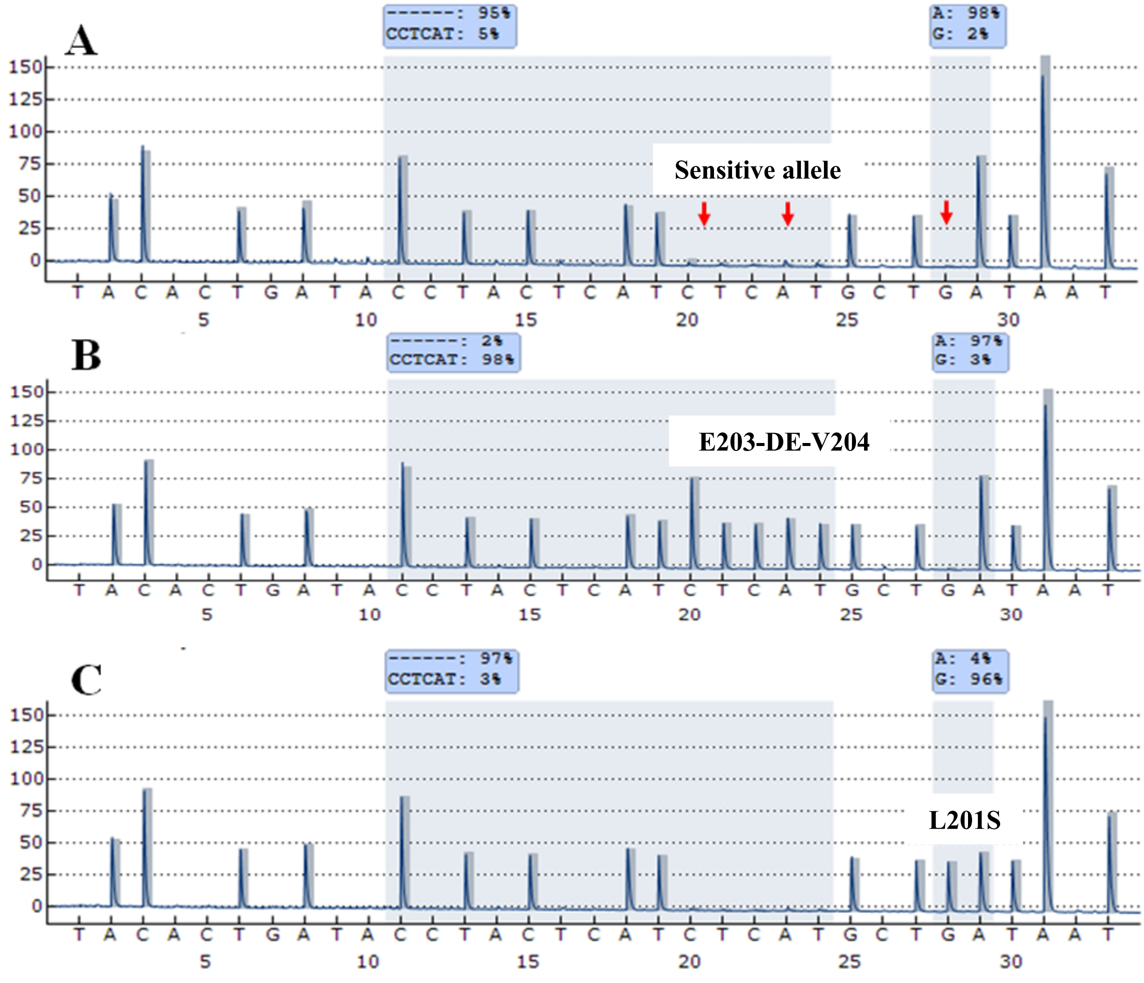

Supplement: S1 Fig — Pyrograms show DNA extract of single sporangia isolates CONI-01 (A), CONI-16 (B) and CONI-38 (C). Nucleotide positions from 11 to 24 represent the variable region to be analysed to detect insertion E203-DE-V204 (B). Nucleotide in position 28 concerns quantification of L201S substitution (C). (PDF) [file pone.0268385.s001.pdf]
