# Supplementary material for: New insights from short and long reads sequencing to explore cytochrome b variants in Plasmopara viticola populations collected from vineyards and related to resistance to complex III inhibitors
Source: PLoS One. 2023 Jan 19;18(1):e0268385. doi: 10.1371/journal.pone.0268385 (PMC9851517; doi:10.1371/journal.pone.0268385)

Fig S2. Pyrograms showing allele quantification of E203-VE-V204 variant from sensitive and cyazofamid-resistant *P. viticola* strains. Pyrograms show DNA extract of single sporangia isolates CONI-01 (A) and CONI-39 (B). Nucleotides positions 8 to 10 represents the variable region to be analysed to detect insertion E203-VE-V204 (B).

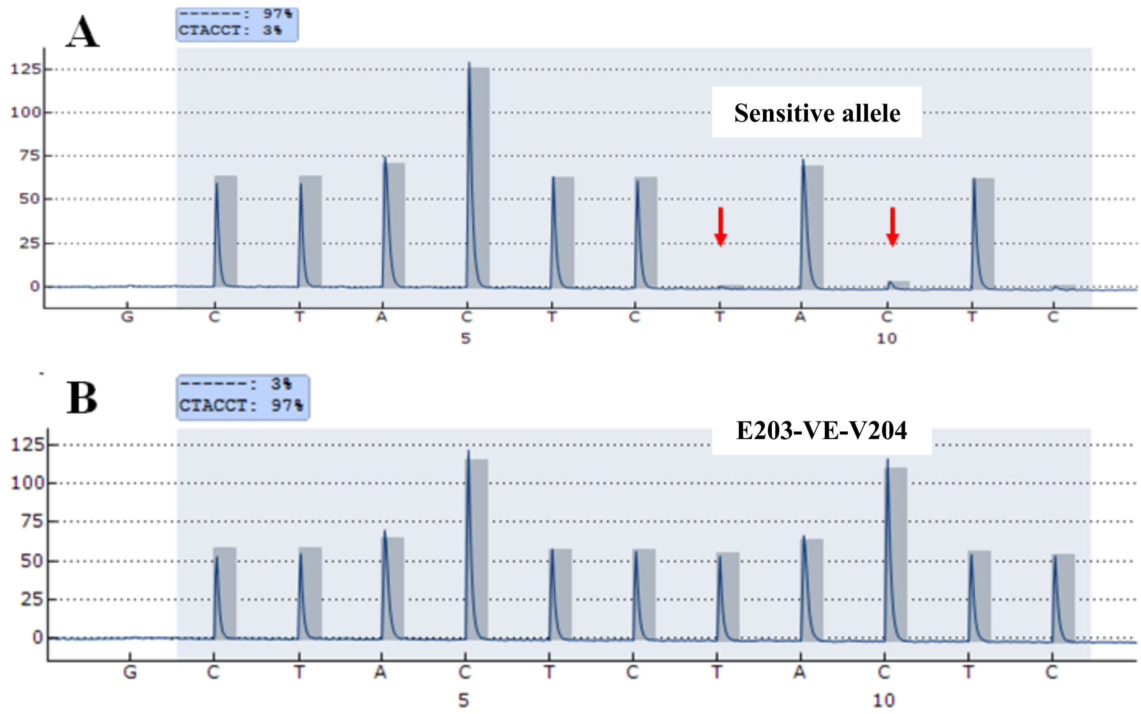

Supplement: S2 Fig — Pyrograms show DNA extract of single sporangia isolates CONI-01 (A) and CONI-39 (B). Nucleotide positions 8 to 10 represent the variable region to be analysed to detect insertion E203-VE-V204 (B). (PDF) [file pone.0268385.s002.pdf]
