# Supplementary material for: New insights from short and long reads sequencing to explore cytochrome b variants in Plasmopara viticola populations collected from vineyards and related to resistance to complex III inhibitors
Source: PLoS One. 2023 Jan 19;18(1):e0268385. doi: 10.1371/journal.pone.0268385 (PMC9851517; doi:10.1371/journal.pone.0268385)

S3 Fig. Locations of the 18 field samples of *P. viticola* studied. The French wine regions where the populations sampled come from are indicated. Samples are identified by the CONI-PX number used in Table 3. The map is designed by @comersis.com.

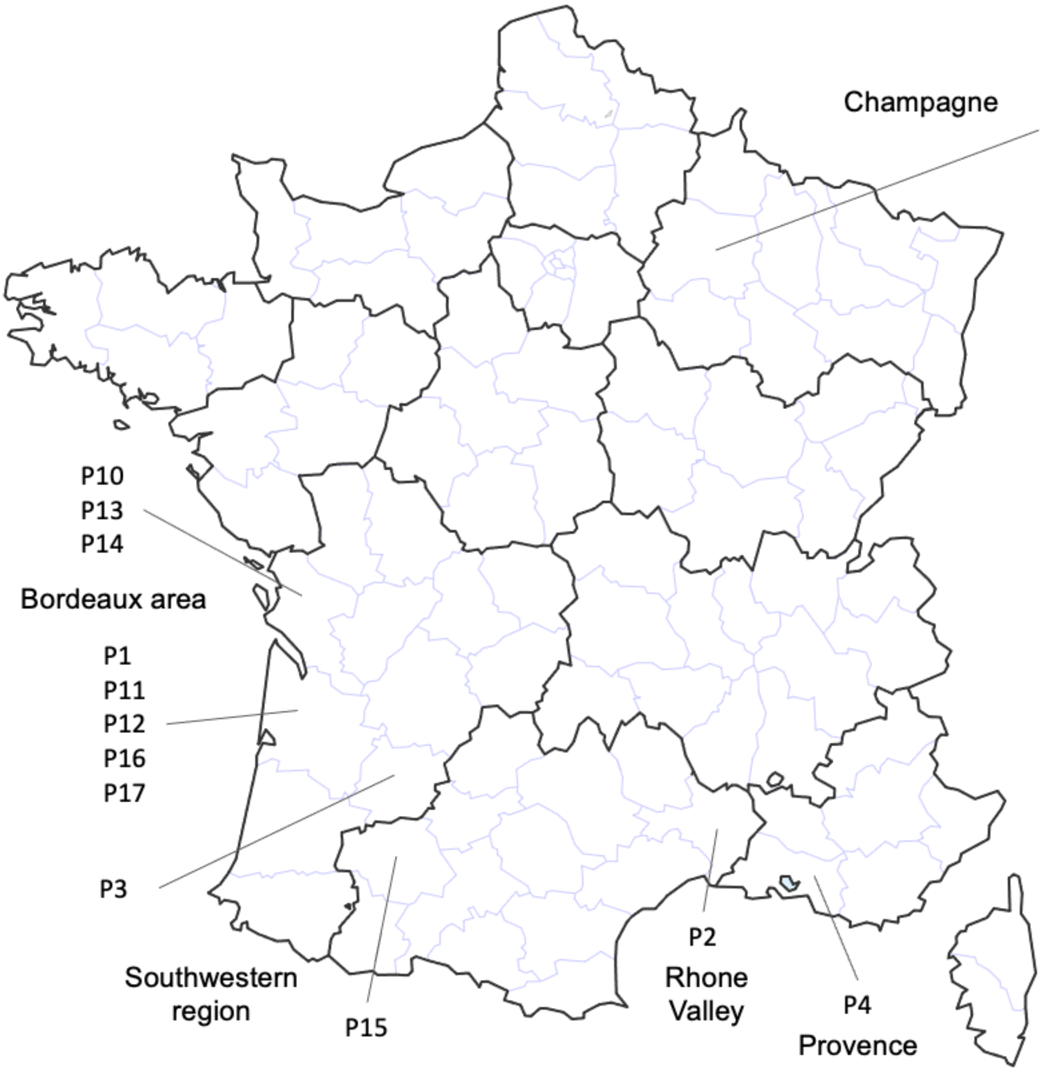

Supplement: S3 Fig — The French wine regions where the populations sampled come from are indicated. Samples are identified by the CONI-PX number used in Table 3. The map is designed by @comersis.com. (PDF) [file pone.0268385.s003.pdf]
