# Supplementary material for: New insights from short and long reads sequencing to explore cytochrome b variants in Plasmopara viticola populations collected from vineyards and related to resistance to complex III inhibitors
Source: PLoS One. 2023 Jan 19;18(1):e0268385. doi: 10.1371/journal.pone.0268385 (PMC9851517; doi:10.1371/journal.pone.0268385)
Supplement: S1 Table — For Ion Torrent data, variants were searched without a priori with Torrent Variant Caller (somatic and low stringency parameters) and only variants above 5% are reported. For ONT data, two dedicated pipelines using either BLAST or Nanopolish were tested. The 3 variants were specifically targeted in all samples. (PDF) [file pone.0268385.s004.pdf]

Table S1: Detection and frequency of E203-DE-V204, E203-VE-V204 and L201S variants observed with short-read (Ion Torrent) and long-read (ONT) sequencing of cyt*b* gene amplified from single sporangia strains. For Ion Torrent data, variants were searched without a priori with Torrent Variant Caller (somatic and low stringency parameters) and only variants above 5% are reported. For ONT data, two dedicated pipelines using either BLAST or Nanopolish were tested. The 3 variants were specifically targeted in all samples.

**Single-sporangia strains tested**	**Sanger genotyped**	**Variant Caller / Ion Torrent sequencing**			**BLAST - Nanopolish / ONT sequencing**		
		E203-DE-V204	E203-VE-V204	L201S	E203-DE-V204	E203-VE-V204	L201S
CONI-01	Wild	-	-	-	0.09 - 0.48	0 - 2	1.19 - 3.58
CONI-08	E203-DE-V204	98.2	-	-	98.02 - 95.81	1.68 - 5.04	0.1 - 3.65
CONI-16	E203-DE-V204	95.2	-	-	95.45 - 94.12	1.44 - 5.31	0 - 4.06
CONI-39	E203-VE-V204	-	92.3	-	1.2 - 4.11	95.91 - 96.92	0.24 - 3.78
CONI-41	E203-VE-V204	-	92.5	-	0.86 - 4.02	96.8 - 97.2	0 - 4.22
CONI-31	L201S	-	-	99.8	0 - 0.76	0.12 - 2	98.87 - 98.58
CONI-38	L201S	-	-	100.0	0 - 0.51	0.23 - 1.78	98.83 - 98.15
